# Some EPR Signals in Tumour Tissue

**DOI:** 10.1038/bjc.1973.145

**Published:** 1973-09

**Authors:** N. J. F. Dodd

## Abstract

Normal and tumour tissues from rats, blood from normal and tumour bearing rats, and normal human blood were examined using the electron paramagnetic resonance (epr) technique. At low temperature a triplet epr signal, which is known to be produced by a NO-haemoprotein complex, was detected in some tumour samples and in decaying normal liver. At room temperature all of the tumour samples examined gave a doublet signal. This signal was also detected in blood but not in other normal tissues. The signal has a g value of 2·0054 ± 0·0002 and a hyperfine splitting of 1·80 ± 0·05 G and is assigned to the ascorbyl free radical. Model experiments suggest that the appearance of detectable concentrations of this radical result from a disturbance of the normal state of the ascorbic acid, dehydroascorbic acid redox system. It was verified that cell division is not responsible for the ascorbyl radical although autolysis may be involved. A possible relationship between the formation of ascorbyl radicals and other paramagnetic species in tumours is discussed.


*Br. J. Cancer* (1973) **28**, 257

### SOME EPR SIGNALS IN TUMOUR TISSUE

N. J. F. DODD

*From the Paterson Laboratories, Christie Hospital and Holt Radium Institute, Manchester M*20 9*BX*

Received 4 April 1973. Accepted 4 June 1973

**Summary.—Normal and tumour tissues from rats, blood from normal and tumour bearing rats, and normal human blood were examined using the electron paramagnetic resonance (epr) technique. At low temperature a triplet epr signal, which is known to be produced by a NO-haemoprotein complex, was detected in some tumour samples and in decaying normal liver. At room temperature all of the tumour samples examined gave a doublet signal. This signal was also detected in blood but not in other normal tissues. The signal has a g value of 2·0054 ± 0·0002 and a hyperfine splitting of 1·80 ± 0·05 G and is assigned to the ascorbyl free radical. Model experiments suggest that the appearance of detectable concentrations of this radical result from a disturbance of the normal state of the ascorbic acid, dehydroascorbic acid redox system. It was verified that cell division is not responsible for the ascorbyl radical although autolysis may be involved. A possible relationship between the formation of ascorbyl radicals and other paramagnetic species in tumours is discussed.**

THE epr technique has been used to study the differences between normal and malignant tissues using lyophilized (Commoner, Townsend and Pake, 1954; Saprin *et al.*, 1966*a*, *b*, *c*; Driscol *et al.*, 1967; Mulay and Mulay, 1967; Wallace *et al.*, 1970), frozen (Nebert and Mason, 1963; Hodgkinson and Cole, 1965; Slater and Cook, 1969) and wet samples (Mallard and Kent, 1966; Swartz, Lewis and Darin, 1971; Swartz, 1972; Duchesne and Van de Vorst, 1970). However, these differences are difficult to interpret, due primarily to a lack of identifiable paramagnetic species. There are 3 notable exceptions to this. The first is a signal with a g value of 2·035 observed in the liver of rats fed with several different carcinogens (Vithayathil, Ternberg and Commoner, 1965). The second is a signal showing a nitrogen triplet hyperfine splitting and seen in frozen samples of a virus-induced reticulum cell sarcoma of the spleen and a neuroblastoma of mice (Brennan, Cole and Singley, 1966), in a hepatoma, sarcoma and Walker carcinoma (Emanuel *et al.*, 1969) and in a human bladder tumour sample (Matsunaga, 1969). The third is a narrow doublet signal reported in low speed cleared sucrose homogenates of mouse melanomata examined at room temperature (Duke, Hourani and Demopoulos, 1967; Duke, 1968). This communication reports the occurrence of a similar doublet signal in wet tissue slices from several rat tumours. The interrelationship of the 3 signals is examined and possible metabolic links are discussed.

### MATERIALS AND METHODS

Slices of normal and malignant tissue weighing 15–30 mg and about 0·5 mm thick were examined at room temperature in flat quartz cells and at low temperature in 3 mm (i.d.) quartz tubes using a Varian E9 epr spectrometer equipped with a variable temperature accessory (Varian E-257). Blood samples were examined at room temperature in a Varian aqueous solution sample cell. Spectra were recorded as first derivatives of the absorption, using 100 kHz modulation, with an amplitude of 0·1–0·5 G. The incident microwave power was 10 mW. Normal and malignant tissues were obtained from male and female Wistar rats of approximately 250 g body weight under ether narcosis. Blood samples were obtained by cardiac puncture. Specimens were stored in covered petri dishes on ice before examination at room temperature and in liquid $N_2$ when required for low temperature examination. The chemicals used were obtained from Koch–Light Laboratories Ltd.

### RESULTS AND DISCUSSION

*The signal.*—A doublet epr signal (Fig. 1) was repeatedly detected in several different rat tumour tissues (Table I) examined at room temperature within minutes of excision. The signal had a g value of $2{\cdot}0054 \pm 0{\cdot}0002$, a hyperfine splitting of $1{\cdot}80 \pm 0{\cdot}05$ G and a line width of $0{\cdot}35 \pm 0{\cdot}05$ G. Microwave power saturation was observed at incident powers greater than 20 mW. Between 3 and 5 samples were taken from each tumour. The magnitude of the signal varied from one area of a tumour to another, in some cases by a factor as great as 10. It was not detected in the apparently normal tissue surrounding the tumour. However, no consistent variation of signal height with distance from the centre of the tumour was observed in different tumours of the same type and age. It is estimated that the free radical concentration of all the tumours examined was between $5 \times 10^{13}$ and $5 \times 10^{14}$ radicals $g^{-1}$. When tumour samples were stored on ice the doublet signal did not change significantly for at least 2 days. However, the signal from tumour slices examined in the flat quartz cell at room temperature, while showing no change for the first 30 min, decayed by about 50% in the next 60 min.

FIG. 1.—Epr signal from 30 mg of tissue freshly excised from a transplanted rat hepatoma. The signal was recorded at room temperature with a modulation amplitude of 0·1 G. The magnetic field increases to the right.

TABLE I.—*Tumour Tissues from Wistar Rats found to Show the Doublet EPR Signal*

| Tumour | Rat: Sex | Rat: Age | Production of tumour | Number of tumours examined |
|---|---|---|---|---|
| Primary hepatoma | ♂ | 8 months | Rats fed diet containing 4-dimethyl-aminoazo-benzene (Baldwin, 1964) | 3 |
| Hepatoma D23 | ♂ | 6–8 weeks | Transplantation. Originally derived from primary hepatoma | 7 |
| Lung tumour | ♂ | 6–8 weeks | I.V. injection of D23 cells, grown in culture | 2 |
| Yoshida (MDMS sensitive) | ♀ | 12 weeks | Transplantation | 10 |
| Yoshida (MDMS resistant) | ♀ | 12 weeks | Transplantation | 10 |
| Walker | ♀ | 12 weeks | Transplantation | 4 |

This decay is about 5 times faster than that reported for the free radical population in normal rat liver (Duchesne and Van de Vorst, 1970).

The doublet signal was not detected in freshly excised samples of liver, heart, spleen, lung, kidney, adrenal, testis or thymus, but was detected in heparinized fresh whole blood from normal and tumour bearing animals as well as in normal human blood. It is therefore not unique to tumours, but is indicative of differences between normal and malignant tissue within, for example, liver. The signal in blood corresponded to a concentration of about $5 \times 10^{13}$ radicals $g^{-1}$. This is very close to the value of $0 \cdot 1 \times 10^{-9}$ mol $g^{-1}$, *i.e.*, $6 \times 10^{13}$ radicals $g^{-1}$, reported previously (Mallard and Kent, 1966) although in that case no hyperfine structure was observed. The contribution made by blood to the signal in tissue is negligible, as demonstrated by the absence of a detectable signal in either blood filled or exsanguinated normal liver. The doublet from blood was localized in the plasma or serum, which gave a signal 5–10 times greater than whole blood. It was undetectable in the cell fraction and on recombining plasma and cells the signal was reduced to the level originally observed in whole blood.

*Nature of the radical.*—The spectral parameters of the doublet are similar to those of the signal observed in mouse melanoma homogenate (Duke *et al.*, 1967; Duke, 1968) and suggest the presence of the ascorbyl radical (Fig. 2). This is the reactive intermediate in the ascorbic acid, dehydroascorbic acid redox system. The assignment is made for the following reasons: (a) The ascorbyl radical, which has recently been shown to exist as the anion with the unpaired electron delocalized over a highly conjugated tricarbonyl system (Laroff, Fessenden and Schuler, 1972), gives a spectrum with a g value of 2·0052, consisting of a doublet of triplets (Lagercrantz, 1964) with splittings of 1·76 G and 0·19 G and an additional doublet splitting of 0·07 G (Laroff *et al.*, 1972). Although it has not been possible to demonstrate any structure in the doublet from tumour tissue, the line width observed does not rule out unresolved fine structure. (b) A solution of ascorbic acid in distilled water gave a signal, showing only the doublet splitting, that was identical to the signals of biological origin. (c) Addition of ascorbic acid to plasma increased the magnitude of the signal without producing any detectable increase in line width. It is therefore believed that the doublet signal detected in tumour tissue and blood is due to the ascorbyl radical, and is referred to as such from now on.

*A possible chemical model.*—The ascorbyl radical can be formed by oxidation of ascorbic acid or reduction of dehydroascorbic acid. The following experiment suggests that it is formed, in tumours, by oxidation. When a slow stream of moist $O_2$ was passed over the tumour slice in the epr cavity the radical concentration increased relative to that in air, whereas flowing $N_2$ over the slice decreased the radical concentration. These changes, which are comparable with those reported for mouse melanoma homogenates (Duke

Ascorbic acid Ascorbyl radical Dehydroascorbic acid

FIG. 2.—The structural formulae of ascorbic acid, the ascorbyl radical and dehydroascorbic acid. The ascorbyl radical is an intermediate in the redox system.

*et al.*, 1967; Duke, 1968) could be produced repeatedly, but both in $O_2$ and in $N_2$ a slow irreversible decay occurred. The reducing agent $H_2S$ rapidly removed the ascorbyl radicals.

Liver slices, which contain a similar concentration of ascorbic acid to the hepatoma or other tumours (Robertson, 1943), failed to show the presence of ascorbyl radicals on storage in air at room temperature or when exposed to a stream of $O_2$ for several hours. Similarly adrenals, which have one of the highest ascorbic acid levels of any tissue, did not show the ascorbyl doublet signal when examined shortly after excision or after storage for several hours on ice. This suggests intracellular control of the oxidation of ascorbic acid.

*A possible control mechanism.*—Glutathione reduces dehydroascorbic acid to ascorbic acid at a pH above 6 (Edgar, 1969, 1970) and may influence the state of the redox system *in vivo.* When tumour slices were soaked for several minutes in saline containing glutathione the ascorbyl radical concentration was reduced, whereas soaking in saline alone had little effect. Addition of glutathione to plasma reduced the ascorbyl radical concentration and the presence of glutathione in erythrocytes may explain the markedly higher concentration of radicals detected in plasma than in whole blood.

*Possible biological models.*—Since liver tumours undergo more rapid cell division than normal liver, it was decided to examine foetal and regenerating liver for the appearance of ascorbyl radicals. Foetal rat liver failed to show the presence of ascorbyl radicals. Liver from normal adult rats was examined following partial hepatectomy. Samples were taken 24 hours after the operation, when DNA synthesis is near maximal in this laboratory strain (O'Connor, 1971), and one week later when new tissue was present. In neither case were ascorbyl radicals detected. It is concluded that the appearance of the radicals in tumour tissue is not due simply to processes associated with cell division.

Tumours outgrowing their blood supply show necrotic foci (van den Brenk, 1969), as do autolysing adrenals. Therefore, rat liver was examined after poisoning and after autolysis. Rats were given an oral dose of 25% $CCl_4$ in liquid paraffin (1 ml $CCl_4$/kg body weight) which was sufficient to produce necrosis within 24 hours (Wigglesworth, 1964). Necrosis was confirmed histologically, but the liver did not show the ascorbyl doublet signal. Liver which was kept at room temperature for 2 days also failed to show the presence of ascorbyl radicals. However, when adrenal slices were kept in the epr cell, ascorbyl radicals were detected after about 60 min at room temperature and 4 hours at 0°C. In contrast, when they were sliced and placed in the cell under $N_2$, ascorbyl radicals were undetectable even after 4 hours at room temperature, although on admission of air or $O_2$ to the cell the ascorbyl radical signal appeared within 20 min. Experiments demonstrated that the appearance of detectable concentrations of ascorbyl radicals in adrenal slices was a result of both autolysis and autoxidation. In liver slices the release of ascorbic acid by autolysis may be insufficient to give a detectable number of ascorbyl radicals on autoxidation. The results suggest that the appearance of relatively high concentrations of ascorbyl radicals in tumour tissues may result from autolytic changes occurring *in vivo.*

*Relationship of the doublet signal to other epr signals in tissues.*—Samples of a Yoshida sarcoma, showing the ascorbyl radical signal at room temperature, showed in addition a 1 : 1 : 1 triplet signal, with a g value of 2·01 and hyperfine splitting of 16–17 G, when examined at − 160°C. This signal is from a NO-haemoprotein complex (Maruyama *et al.*, 1971). Several samples from different areas of a Walker tumour all showed the ascorbyl radical signal but only those from the dark red, blood filled areas showed the NO-haemoprotein complex signal. Since degenerating normal tissue also shows this signal (Muruyama *et al.*, 1971) rat liver was examined after storage for 2 days at room temperature. The NO-haemoprotein complex was detected but not the ascorbyl radical. In contrast, the singlet at $g=2{\cdot}035$, from $NO\text{-}Fe^{2+}$ complexes of thiol containing non-haeme proteins, which has been reported in the livers of carcinogen fed rats before the appearance of tumours (Commoner *et al.*, 1970; Vithayathil *et al.*, 1965; Woolum and Commoner, 1970), was not detected in any of the tissues examined.

Paramagnetic NO-protein complexes and ascorbyl radicals are not always observed in the same tissue, but their formation *in vitro* is linked. Ascorbic acid promotes the formation of NO-protein complexes by reaction with nitrites to form NO and ascorbyl radicals (Woolum, Tiezzi and Commoner, 1968; Maruyama *et al.*, 1971). Ascorbic acid also blocks the formation of carcinogenic nitroso-compounds by scavenging nitrites *in vitro* (Mirvish *et al.*, 1972) and possibly *in vivo* (Kamm *et al.*, 1973). The formation of ascorbyl radicals in tumour tissue may result from reactions of ascorbic acid, some of which give NO, which in turn may be responsible for formation of the paramagnetic NO-complexes observed.

The author wishes to thank Drs A. W. Craig and P. J. O'Connor and in particular Dr M. Ebert for helpful discussions during the preparation of the manuscript, and Mr R. Thompson for expert technical assistance. The hepatomata were kindly supplied by Dr R. W. Baldwin, University of Nottingham. This work was supported by the Medical Research Council and the Cancer Research Campaign.

### REFERENCES

BALDWIN, R. W. (1964) Modification of Cell Antigens during Aminoazo Dye Carcinogenesis in Rat Liver. *Br. J. Cancer*, **18**, 285.

BRENNAN, M. J., COLE, T. & SINGLEY, J. A. (1966) A Unique Hyperfine ESR Spectrum in Mouse Neoplasms Analysed by Computer Simulation. *Proc. Soc. exp. Biol. Med.*, **123**, 715.

COMMONER, B., TOWNSEND, J. & PAKE, G. E. (1954) Free Radicals in Biological Materials. *Nature, Lond.*, **174**, 689.

COMMONER, B., WOOLUM, J. C., SENTURIA, B. H. & TERNBERG, J. L. (1970) The Effects of 2-acetyloaminofluorene and Nitrite on Free Radicals and Carcinogenesis in Rat Liver. *Cancer Res.*, **30**, 2091.

DRISCOL, D. H., DETTMER, C. M., WALLACE, J. D. & NEAVES, A. (1967) Variation of ESR Signal Amplitude with Duration of Tumour Growth. *Curr. Mod. Biol.*, **1**, 275.

DUCHESNE, J. & VAN DE VORST, A. (1970) Free Radicals in Normal and Pathological Surviving Tissues. *Bull. Acad. r. Belg, (classe des sciences)*. **56**, 433.

DUKE, P. S. (1968) Relation of Melanoma Homogenate and Ascorbate Solution EPR Doublets. *Exp. & Mol. Pathol.*, **8**, 112.

DUKE, P. S., HOURANI, B. J. & DEMOPOULOS, H. B. (1967) Study of S91 Mouse Melanomas by EPR Spectroscopy and Tissue Culture. II. Effects of Penicillamine on EPR Signals and on Growth *in vitro*. *J. natn. Cancer Inst.*, **39**, 1141.

EDGAR, J. A. (1969) Is Dehydroascorbic Acid an Inhibitor in the Regulation of Cell Division in Plants and Animals? *Experientia*, **25**, 1214.

EDGAR, J. A. (1970) Dehydroascorbic Acid and Cell Division. *Nature, Lond.*, **227**, 24.

EMANUEL, N. M., SAPRIN, A. N., SHABALKIN, V. A., KOZLOVA, L. E. & KRUGLYAKOVA, K. E. (1969) Detection and Investigation of a New Type of ESR Signal Characteristic of some Tumour Tissues. *Nature, Lond.*, **222**, 165.

HODGKINSON, C. P. & COLE, T. (1965) Electron Spin Resonance Spectra in Normal and Malignant Human Tissue. *Obstet. Gynecol.*, **25**, 411.

KAMM, J. J., DASHMAN, T., CONNEY, A. H. & BURNS, J. J. (1973) Protective Effect of Ascorbic Acid on Hepatoxicity Caused by Sodium Nitrite plus Aminopyrine. *Proc. natn. Acad. Sci. U.S.A.*, **70**, 747.

LAGERCRANTZ, C. (1964) Free Radicals in the Autooxidation of Ascorbic Acid. *Acta chem. scand.*, **18**, 562.

LAROFF, G. P., FESSENDEN, R. W. & SCHULER, R. H. (1972) ESR Spectra of Radical Intermediates in the Oxidation of Ascorbic Acid and Related Substances. *J. Am. chem. Soc.*, **94**, 9062.

MALLARD, J. R. & KENT, M. (1966) Electron Spin Resonance in Surviving Rat Tissues. *Nature, Lond.*, **210**, 588.

MARUYAMA, T., KATAOKA, N., NAGASE, S., NAKADA, H., SATO, H. & SASAKI, H. (1971) Identification of Three Line Electron Spin Resonance Signal and its Relationships to Ascites Tumours. *Cancer Res.*, **31**, 179.

MATSUNAGA, J. (1969) Studies on Free Radicals in Tumour Tissue of the Urinary Bladder. 1. Free Radicals in Human Normal Bladder Mucosa and Transitional Cell Carcinoma of the Urinary Bladder Analysed with ESR. *Jap. J. Urol.*, **60**, 214.

MIRVISH, S. S., WALLCAVE, L., EAGEN, M. & SHUBIK, P. (1972) Ascorbate–nitrite Reaction; Possible Means of Blocking for Formation of Carcinogenic N-nitroso Compounds. *Science, N.Y.*, **177**, 65.

MULAY, I. L. & MULAY, L. N. (1967) Magnetic Susceptibility and Electron Spin Resonance Absorption Spectra of Mouse Melanomas S91 and S91A. *J. natn. Cancer Inst.*, **39**, 735.

NEBERT, D. W. & MASON, H. S. (1963) An Electron Spin Resonance Study of Neoplasms. *Cancer Res.*, **23**, 833.

O'Connor, P. J. (1971) Changes in Alkaline Deoxyribonuclease Activity in Rat Liver Following Partial Hepatectomy. *Biochem. biophys. acta*, **238**, 186.

Robertson, W. V. B. (1943) Ascorbic Acid Content of Tumours and Homologous Normal Tissues. *J. natn. Cancer Inst.*, **4**, 321.

Saprin, A. N., Klochko, E. V., Kruglyakova, K. E., Chibrikin, V. M. & Emanuel, N. M. (1966*a*) Kinetic Patterns of Change in the Content of Free Radicals on Malignant Growth and the Effects of Inhibitors of Radical Processes. *Biofizika*, **11**, 443.

Saprin, A. N., Minenkova, E. A., Nagler, L. G., Koperina, E. V., Kruglyako, S. A., Kruglyakova, K. E., Vermel, E. M. & Emanuel, N. M. (1966*b*) Kinetics of Change in the Content of Free Radicals with Development of Ascites Sarcoma 37. *Biofizika*, **11**, 616.

Saprin, A. N., Nagler, L. G., Koperina, E. V., Kruglyakova, K. E. & Emanuel, N. M. (1966*c*) Kinetics of Change in the Content of Free Radicals in the Blood and Organs of Mice with Experimental Leukaemia. II. *Biofizika*, **11**, 706.

Slater, T. F. & Cook, J. W. R. (1969) Electron Spin Resonance Studies on Normal and Malignant Human Tissues and in Normal and Damaged Rat Liver. In *Cytology Automation.* Ed. D. M. D. Evans. Edinburgh: Livingstone. p. 108.

Swartz, H. M., Lewis, J. D. & Darin, J. C. (1971) *ESR Studies of Tissue Viability.* Proc. First European Biophysics Congress, Baden, Austria. Ed. E. Broda, A. Locker and H. Springer-Lederer. Wiener Medizinschen Akademie. p. 557.

Swartz, H. M. (1972) Cells and Tissues. In *Biological Applications of Electron Spin Resonance.* Ed. H. M. Swartz, J. R. Bolton and D. C. Borg. New York: Wiley-Interscience. Ch. 4, p. 155.

Van den Brenk, H. A. S. (1969) The Oxygen Effect in Radiation Therapy. In *Current Topics in Radiation Research.* Eds. M. Ebert and A. Howard. Amsterdam: North Holland Publishing Co. **5**, p. 199.

Vithayathil, A. J., Ternberg, J. L. & Commoner, B. (1965) Changes in Electron Spin Resonance Signals of Rat Liver during Chemical Carcinogenesis. *Nature, Lond.*, **207**, 1246.

Wallace, J. D., Driscoll, D. H., Kalomiris, C. G. & Neaves, A. (1970) A Study of Free Radicals Occurring in Tumorous Female Breast Tissue and their Implication to Detection. *Cancer, N.Y.*, **25**, 1087.

Wigglesworth, J. S. (1964) The Use of the Osmium Ethyl Gallate Technique in the Study of Carbon Tetrachloride Liver Injury. *J. Path. Bact.*, **87**, 333.

Woolum, J. C. & Commoner, B. (1970) Isolation and Identification of a Paramagnetic Complex from the Livers of Carcinogen-treated Rats. *Biochem. biophys. acta*, **201**, 131.

Woolum, J. C., Tiezzi, E. & Commoner, B. (1968) Electron Spin Resonance of Iron–Nitric Oxide Complexes with Amino Acids, Peptides and Proteins. *Biochem. biophys. acta*, **160**, 311.


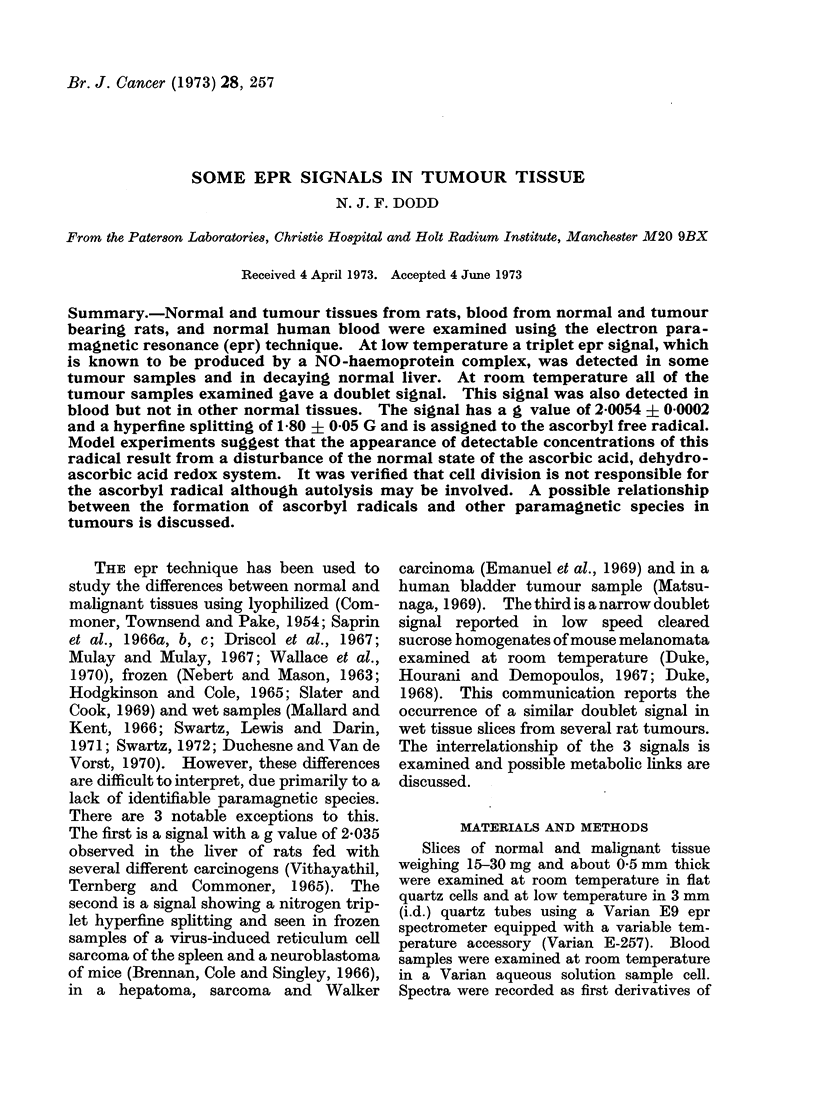

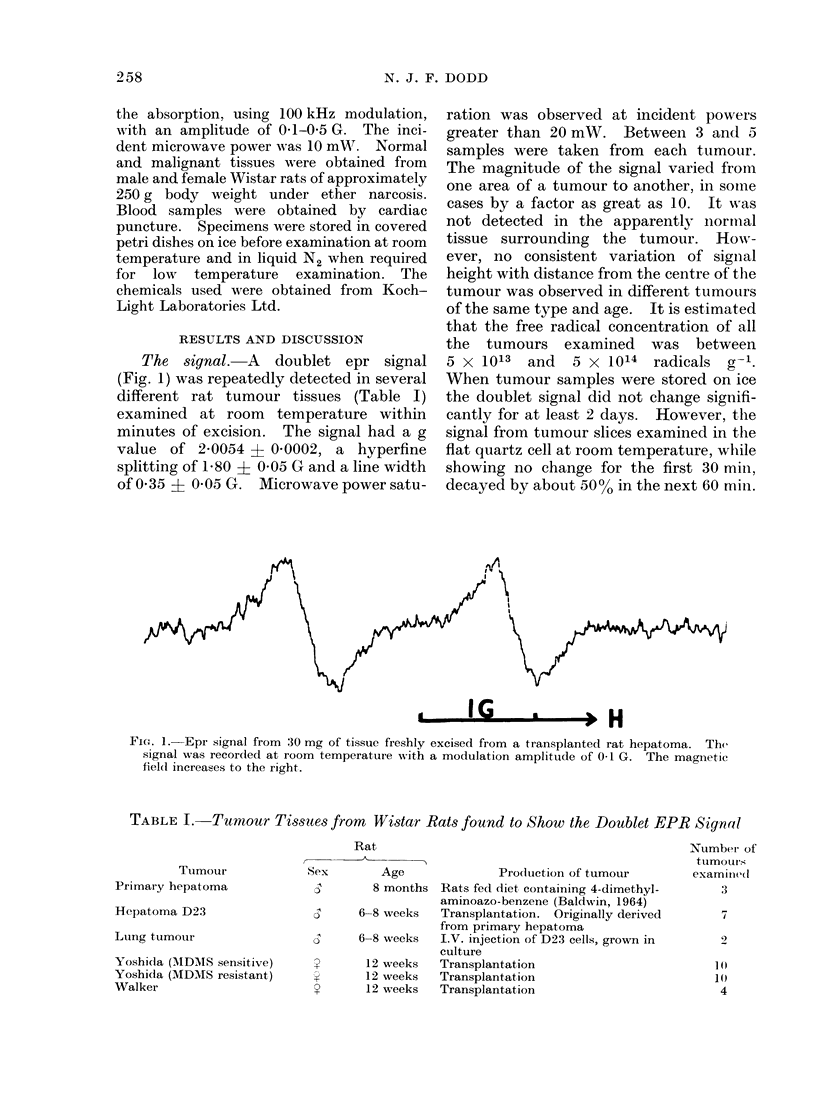

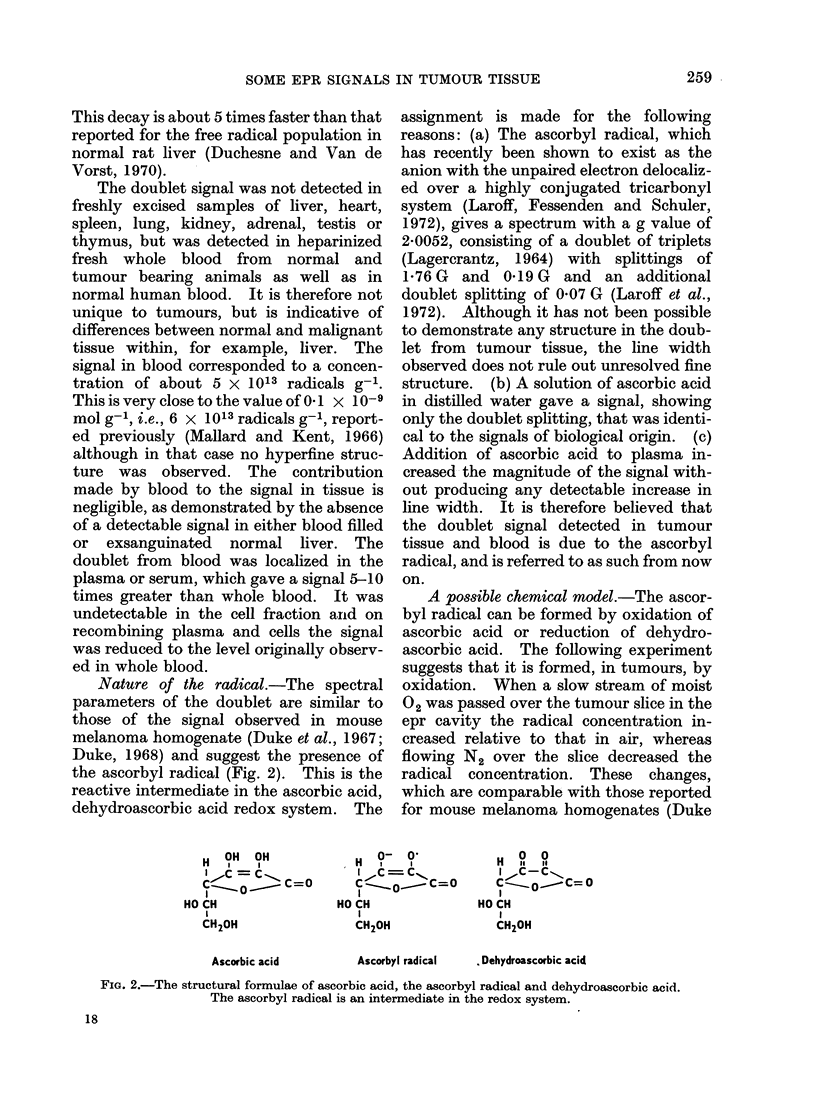

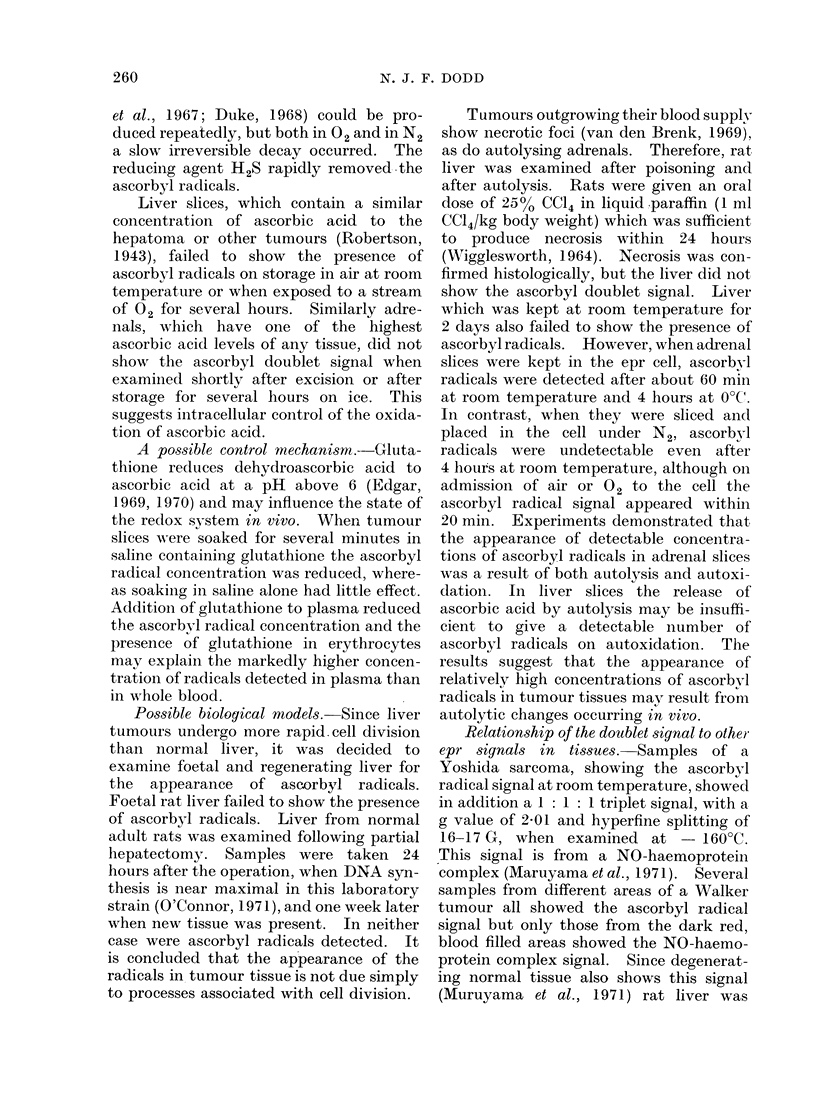

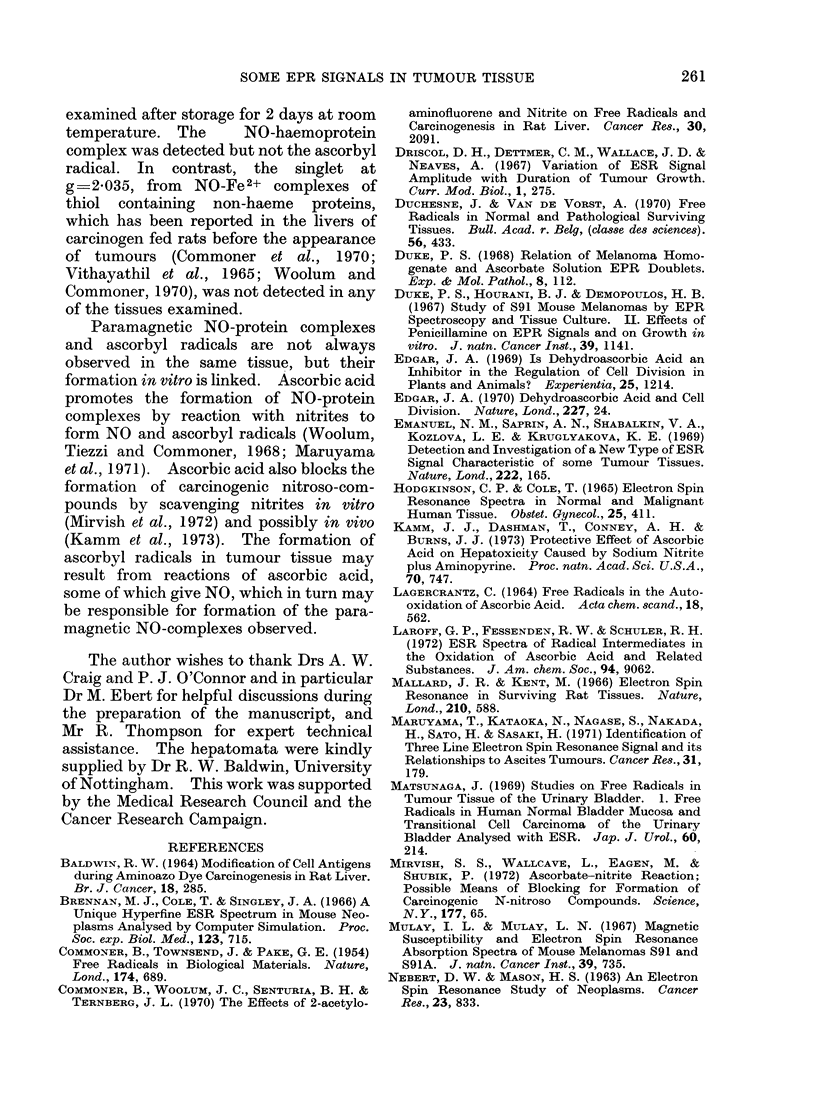

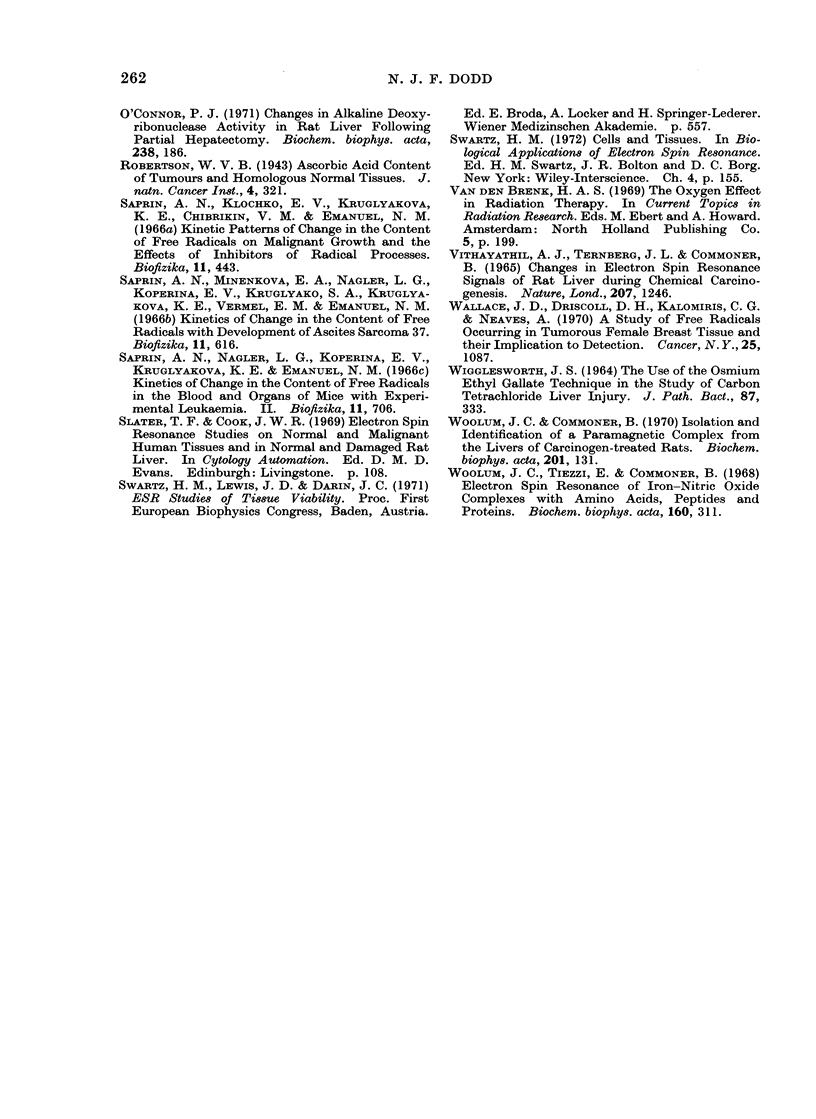


## References

[OCR_00470] BALDWIN R. W. (1964). MODIFICATION OF CELL ANTIGENS DURING AMINOAZO DYE CARCINOGENESIS IN RAT LIVER.. Br J Cancer.

[OCR_00475] Brennan M. J., Cole T., Singley J. A. (1966). A unique hyperfine ESR spectrum in mouse neoplasms analyzed by computer simulation.. Proc Soc Exp Biol Med.

[OCR_00481] COMMONER B., TOWNSEND J., PAKE G. E. (1954). Free radicals in biological materials.. Nature.

[OCR_00486] Commoner B., Woolum J. C., Senturia B. H., Ternberg J. L. (1970). The effects of 2-acetylaminofluorene and nitrite on free radicals and carcinogenesis in rat liver.. Cancer Res.

[OCR_00494] Driscoll D. H., Dettmer C. M., Wallace J. D., Neaves A. (1967). Variation of ESR signal amplitude with duration of tumor growth.. Curr Mod Biol.

[OCR_00511] Duke P. S., Hourani B. T., Demopoulos H. B. (1967). Study of S91 mouse melanomas by electron paramagnetic resonance (EPR) spectroscopy and tissue culture. II. Effects of penicillamine on EPR signals and on growth in vitro.. J Natl Cancer Inst.

[OCR_00523] Edgar J. A. (1970). Dehydroascorbic acid and cell division.. Nature.

[OCR_00518] Edgar J. A. (1969). Is dehydroascorbic acid an inhibitor in the regulation of cell division in plants and animals?. Experientia.

[OCR_00527] Emanuel N. M., Saprin A. N., Shabalkin V. A., Kozlova L. E., Krugljakova K. E. (1969). Detection and investigation of a new type of ESR signal characteristic of some tumour tissues.. Nature.

[OCR_00539] Kamm J. J., Dashman T., Conney A. H., Burns J. J. (1973). Protective effect of ascorbic acid on hepatotoxicity caused by sodium nitrite plus aminopyrine.. Proc Natl Acad Sci U S A.

[OCR_00551] Laroff G. P., Fessenden R. W., Schuler R. H. (1972). The electron spin resonance spectra of radical intermediates in the oxidation of ascorbic acid and related substances.. J Am Chem Soc.

[OCR_00557] Mallard J. R., Kent M. (1966). Electron spin resonance in surviving rat tissues.. Nature.

[OCR_00562] Maruyama T., Kataoka N., Nagase S., Nakada H., Sato H., Sasaki H. (1971). Identification of three-line electron spin resonance signal and its relationship to ascites tumors.. Cancer Res.

[OCR_00577] Mirvish S. S., Wallcave L., Eagen M., Shubik P. (1972). Ascorbate-nitrite reaction: possible means of blocking the formation of carcinogenic N-nitroso compounds.. Science.

[OCR_00590] NEBERT D. W., MASON H. S. (1963). AN ELECTRON SPIN RESONANCE STUDY OF NEOPLASMS.. Cancer Res.

[OCR_00597] O'Connor P. J. (1971). Changes in alkaline deoxyribonuclease activity in rat liver following partial hepatectomy.. Biochim Biophys Acta.

[OCR_00608] Saprin A. N., Klochko E. V., Chibrikin V. M., Krugliakova K. E., Emanuel' N. M. (1966). Kineticheskie zakonomernosti izmeneniia soderzhaniia svobodnykh radikalov pri zlokachestvennom roste i deistvii ingibitorov radikal'nykh protsessov.. Biofizika.

[OCR_00619] Saprin A. N., Minenkova E. A., Nagler L. G., Koperina E. V., Krugliak S. A., Krugliakova K. E., Vermel' E. M., Emanuel' N. M. (1966). 3. Kinetika izmeneniia soderzhaniia svobodnykh radikalov pri razvitii astsitnoi sarkomy 37.. Biofizika.

[OCR_00659] Vithayathil A. J., Ternberg J. L., Commoner B. (1965). Changes in electron spin resonance signals of rat liver during chemical carcinogenesis.. Nature.

[OCR_00672] WIGGLESWORTH J. S. (1964). THE USE OF THE OSMIUM-ETHYL GALLATE TECHNIQUE IN THE STUDY OF CARBON TETRACHLORIDE LIVER INJURY.. J Pathol Bacteriol.

[OCR_00665] Wallace J. D., Driscoll D. H., Kalomiris C. G., Neaves A. (1970). A study of free radicals occurring in tumorous female breast tissue and their implication to detection.. Cancer.

[OCR_00678] Woolum J. C., Commoner B. (1970). Isolation and identification of a paramagnetic complex from the livers of carcinogen-treated rats.. Biochim Biophys Acta.

[OCR_00684] Woolum J. C., Tiezzi E., Commoner B. (1968). Electron spin resonane of iron-nitric oxide complexes with amino acids, peptides and proteins.. Biochim Biophys Acta.

